# Isoform switching leads to downregulation of cytokine producing genes in estrogen receptor positive breast cancer

**DOI:** 10.3389/fgene.2023.1230998

**Published:** 2023-10-13

**Authors:** Mohammad Shahbaz Khan, Waqar Hanif, Nada Alsakhen, Basit Jabbar, Israa M. Shamkh, Ahad Amer Alsaiari, Mazen Almehmadi, Saad Alghamdi, Afnan Shakoori, Dunia A. Al Farraj, Saeedah Musaed Almutairi, Yasser Hussein Issa Mohammed, Amr S. Abouzied, Aziz-Ur Rehman, Bader Huwaimel

**Affiliations:** ^1^ Children’s National Hospital, Washington, DC, United States; ^2^ Department of Bioinformatics, Department of Sciences, School of Interdisciplinary Engineering & Science (SINES), National University of Sciences and Technology (NUST), Islamabad, Pakistan; ^3^ Department of Chemistry, Faculty of Science, The Hashemite University, Zarqa, Jordan; ^4^ Centre of Excellence in Molecular Biology, University of the Punjab, Lahore, Pakistan; ^5^ Chemo and Bioinformatics Lab, Bio Search Research Institution, Giza, Egypt; ^6^ Department of Clinical Laboratory Sciences, College of Applied Medical Sciences, Taif University, Taif, Saudi Arabia; ^7^ Laboratory Medicine Department, Faculty of Applied Medical Sciences, Umm Al-Qura University, Makkah, Saudi Arabia; ^8^ Department of Botany and Microbiology, College of Science, King Saud University, Riyadh, Saudi Arabia; ^9^ Department of Biochemistry, Faculty of Applied Science, University of Hajjah, Hajjah, Yemen; ^10^ Department of Pharmaceutical Chemistry, College of Pharmacy, University of Hail, Hail, Saudi Arabia; ^11^ Department of Pharmaceutical Chemistry, National Organization for Drug Control and Research (NOD CAR), Giza, Egypt; ^12^ Keystone Pharmacogenomics LLC, Bensalem, PA, United States; ^13^ Medical and Diagnostic Research Center, University of Hail, Hail, Saudi Arabia

**Keywords:** estrogen receptor, breast cancer, isoform switching, differential expression, functional enrichment, cytokine, immunosuppression, CXCR chemokine receptor

## Abstract

**Objective:** Estrogen receptor breast cancer (BC) is characterized by the expression of estrogen receptors. It is the most common cancer among women, with an incidence rate of 2.26 million cases worldwide. The aim of this study was to identify differentially expressed genes and isoform switching between estrogen receptor positive and triple negative BC samples.

**Methods:** The data were collected from ArrayExpress, followed by preprocessing and subsequent mapping from HISAT2. Read quantification was performed by StringTie, and then R package ballgown was used to perform differential expression analysis. Functional enrichment analysis was conducted using Enrichr, and then immune genes were shortlisted based on the ScType marker database. Isoform switch analysis was also performed using the IsoformSwitchAnalyzeR package.

**Results:** A total of 9,771 differentially expressed genes were identified, of which 86 were upregulated and 117 were downregulated. Six genes were identified as mainly associated with estrogen receptor positive BC, while a novel set of ten genes were found which have not previously been reported in estrogen receptor positive BC. Furthermore, alternative splicing and subsequent isoform usage in the immune system related genes were determined.

**Conclusion:** This study identified the differential usage of isoforms in the immune system related genes in cancer cells that suggest immunosuppression due to the dysregulation of CXCR chemokine receptor binding, iron ion binding, and cytokine activity.

## Introduction

Breast cancer (BC) is one of the most commonly diagnosed global malignancies and is a leading cause of mortality among women. BC is a heterogeneous disease involving multiple environmental and genetic factors such as age, hormones, unhygienic diet, or toxic environmental exposure. The BRCA1 and BRCA2 tumor suppressor genes play a significant role in BC development (Li et al., 2017). Despite advances in treatments like chemotherapy, endocrine therapy, and human epidermal growth factor receptor-2 (HER2)-targeted therapy, the chance of relapse and BC metastasis remains a great challenge (Zhu and Yu, 2022). BC is a global health challenge as the most commonly diagnosed cancer, with an estimated incidence of 2.26 million cases worldwide according to GLOBOCAN 2020 global cancer statistics. The reported BC incidence rate is higher in Asia at 45.4% (Sung et al., 2021). There are different types of BC, depending on which cells in the breast become cancerous. Estrogen receptor positive (ERP) and triple-negative BC (TNBC) are the most aggressive types of BC. ERP BC is characterized by the presence of estrogen receptors (ERs) on tumor cells that help them grow and proliferate rapidly based on estrogen fueling. It is the largest subtype of BC as it involves the expression and activity of the estrogen receptor. It is estimated that approximately 80% of BCs are ERP (Lamb et al., 2019). TNBC is defined as a type of BC with a negative expression of ER, progesterone receptor (PR), and HER2. The mortality rate of TNBC is higher because of its high invasiveness and because approximately 46% of TNBC patients are more likely to have distant metastasis (Yin et al., 2020).

The ERP BC microenvironment (BCM) consists of immune cells, fibroblasts, adipocytes, mesenchymal stem cells, extracellular matrix, and tumor-associated macrophages (TAMs) (Munir et al., 2021). During breast tumorigenesis, tumor cells escape the immune surveillance by modifying surface antigens and altering their surrounding environment (Segovia-Mendoza and Morales-Montor, 2019). Chemokine, a family of signaling proteins, functions to induce leukocyte migration. Chemokine CC receptor type 5 (CCR5) is a cell surface receptor that has a high affinity for chemotropic cytokines called chemokines. A 32-bp deletion in this receptor (CCR5Δ32) results in a non-functional and deformed receptor which, in turn, results in the activation and invasion of immune cells at the site of tumorigenesis and ultimately leads to its progression (Fatima et al., 2019). In mammalian cells, alternative splicing (AS) is a key mechanism of gene expression regulation. AS occurs when intron and exon elements become rearranged by splicing at different splice-sites, resulting in multiple RNA transcripts. AS regulation is influenced by multiple factors such as cancer or other diseases (Vitting-Seerup and Sandelin, 2017). It occurs when there is differential usage of gene transcripts between different conditions (Baralle and Giudice, 2017). Thus, gene expression should be analyzed at the isoform level because isoform switching (IS) with predicted functional consequences is more common and important in dysfunctional cells (Kahraman et al., 2020).

RNA sequencing (RNA-seq) is a proven quantitative tool for the expression estimation of cells and facilitates the detection and identification of novel transcripts generated by AS. This study identified differential isoform usage (DIU) across conditions (ERP vs. TNBC) in immune system-related genes that may assist targeted therapies for ERP BC. Identifying novel biomarkers and isoform switching may pave the way for the early detection and successful treatment of ERP BC (Chen et al., 2022).

## Methodology

### Overview of the protocol

The data were collected from ArrayExpress: E-MTAB-4993. Further processing and analysis were performed by RNA-seq analysis consisting of preprocessing, mapping, quantifying, and differential expression analysis (DEA) methods (Costa-Silva et al., 2017). Isoform switching and DIU were ultimately detected in immune system-related dysregulated genes in ERP vs. TNBC. This study considered two biological conditions of BC, ERP, and TNBC. The raw data comprised 63 samples (ERP = 51, TNBC = 12).

### RNA-seq data preprocessing and mapping

The data obtained from ArrayExpress were in the form of raw reads and required preprocessing and quality control to reduce noise by trimming poor quality reads, adaptors, and primers. The first step in data preprocessing was quality assessment, which was performed by the FastQC tool to generate individual quality reports for each sample (v0.11.9) (Rostovskaya et al., 2022). The fastp tool (v0.20.0) was then used to trim poor quality reads to remove primer and adaptor content, resulting in filtered reads (Chen et al., 2018). These filtered reads then underwent quality check analysis by FastQC. Next, the filtered reads were used as input in the HISAT2 tool (v2.1.0) for mapping against the reference genome of *Homo sapiens* (GRCh38) (Kim et al., 2015). This generated files in SAM (sequence alignment map) format which contained aligned reads. Mapping rates indicative of the quality of RNA sequencing are presented in Supplementary File 6.

### Read quantification and DEA

Before read quantification, the SAM files were first converted to BAM (binary alignment map) format, which is the compressed and binary format of aligned reads, using Samtools (v1.16) (Danecek et al., 2021). Next, BamUtil (v1.0.15) was used to remove duplicates (deduplication) from mapped reads (Jun et al., 2017). The quantification of deduplicated sorted reads was then performed using StringTie (v2.2.0) (Shumate et al., 2022) in three steps. In the first step, the StringTie assembler was employed to assemble the aligned reads of each sample into a transcriptome. In the second step, the full set of transcriptome assemblies was passed to the StringTie merge module to merge the genomic features among all samples to create a consistent set of transcripts across all samples. In the final step, this merged assembly was used to estimate the transcript abundances (Pertea et al., 2016). The identification of differentially expressed genes (DEGs) and their enrichment analysis offers biological insights into the processes that are affected by certain conditions (Frazee et al., 2015). R package ballgown (v3.15) was used to perform differential gene expression (DGE) analysis of all the transcripts and abundances in ERP vs. TNBC (Frazee et al., 2014). Criteria of a *p*-value less than 0.05 and log2 FoldChange value of <1.5 and >1.5 were used to identify biologically and statistically significant DEGs in ERP vs. TNBC. DEGs were graphically represented by the volcano plot (Nisar et al., 2021).

### Functional enrichment analysis

For Gene Ontology (GO) and pathway enrichment analysis, the Enrichr package was used (Xie et al., 2021). The analysis of both up- and downregulated DEGs was performed separately. The plotEnrich () function was used to plot bar charts of biological processes (BP), molecular functions (MF), cellular components (CC), and KEGG pathways. The results were ordered according to *p*-value.

### Identification of immune system-related genes

The ScType cell marker database was used to filter the genes involved in immune functions (regulation of immune cells through signaling pathway and immune response against tumors) from the DEGs identified in the previous step (Gonzalez et al., 2018; Ianevski et al., 2022). Genes common to the DEGs set and the ScType database (immune system) were selected for further analysis.

### Identification of isoform switching in DEGs

Isoform switch analysis was performed to identify transcript-level expression profiles between ERP and TNBC to detect potential functional consequences resulting from isoform switch. The IsoformSwitchAnalyzeR package (v1.16.0) was used for this analysis (Vitting-Seerup and Sandelin, 2017). The package's input was the quantification files from StringTie, transcript files, a file containing merged annotations of all samples, and a design file containing sample IDs and relevant condition status. The IsoformSwitchTestDEXSeq () function was used to identify DIU based on differential isoform (dIF) cutoff. A dIF criteria of 0.1 was used to find the relative abundances of all isoforms of a gene between two sample groups, and gene ExpressionCutoff of 0.5 was applied. The open reading frames (ORF) were analyzed using the analyzeORF() function, where the longest orfMethod was selected in order to shortlist only long ORFs due to their functional importance. The longest ORFs were then extracted using the extractSequence () function that outputs two files—one containing nucleotide sequences and the other including protein sequences. The functional consequences of ORFs were identified in order to add functional knowledge to the transcripts. Four types of functional consequences were identified for ORFs: coding potential, protein domains, signal peptides, and intrinsically disordered regions (IDRs). The coding potential of the genes was identified through the CPC2 tool that takes nucleotide sequences as an input. The Pfam tool was used to predict protein domains. The signal peptides of ORFs were identified through SignalP, whereas intrinsically disordered regions (IDRs) were predicted by the IUPred3 tool. To assign the predicted functional consequences to the transcripts, R package was employed using functions such as analyzeCPC2 (), analyzeSignalP (), analyzePFAM (), and analyzeIUPred2A (). Moreover, the switchPlot () function was used to plot the shortlisted immune system-related genes that were dysregulated in ERP BC.

## Results

### Identification of upregulated and downregulated genes

**FIGURE 1:**
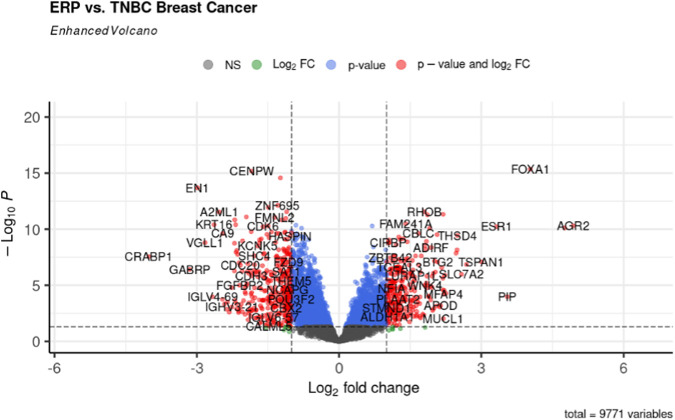
Volcano plot for DEGs. The red dots represent up- (right) and downregulated (left) DEGs. Upregulated genes having logFC >1.5 and *p*-value <0.05 can be seen on the right of the plot; downregulated genes having logFC < −1.5 and *p*-value <0.05 can be seen on the left of the plot.

The gene expression profiling of 63 samples (ERP = 51, TNBC = 12) by ballgown R/Bioconductor identified 15,947 DEGs between ERP and TNBC tissue samples. Genes with no annotation were filtered out, with 9,771 DEGs remaining. Using DEA, 86 genes were identified as upregulated (logFC >1.5 and *p*-value <0.05) and 117 were downregulated (logFC < −1.5 and *p*-value <0.05) (Figure 1). The top 10 upregulated DEGs were FOXA1, RHOB, AR, CMBL, AGR2, ESR1, TFF3, SYBU, CBLC, and DNALI1 (Table 1; Supplementary Information S1); the top 10 downregulated DEGs were CENPW, EN1, A2ML1, TMSB15A, FOXC1, KRT16, SLC7A5, CDK6, MELTF, and CA9 (Table 2; Supplementary Information S2).

**TABLE 1:** Top 10 differentially expressed upregulated genes.

Gene name	*p*-value	log2FoldChange	Expression
FOXA1	4.44E-16	4.03	Up
RHOB	2.75E-12	1.79	Up
AR	4.69E-12	2.19	Up
CMBL	5.16E-12	1.86	Up
AGR2	4.93E-11	4.93	Up
ESR1	5.81E-11	3.32	Up
TFF3	7.72E-11	4.75	Up
SYBU	8.24E-11	1.91	Up
CBLC	2.37E-10	1.67	Up
DNALI1	3.04E-10	2.06	Up

**TABLE 2:** Top 10 differentially expressed downregulated genes.

Gene name	*p*-value	log2FoldChange	Expression
CENPW	6.66E-16	−1.84	Down
EN1	2.06E-14	−2.98	Down
A2ML1	2.95E-12	−2.52	Down
TMSB15A	8.07E-12	−1.95	Down
FOXC1	1.47E-11	−2.20	Down
KRT16	3.93E-11	−2.63	Down
SLC7A5	3.98E-11	−2.18	Down
CDK6	5.90E-11	−1.60	Down
MELTF	1.66E-10	−1.85	Down
CA9	2.41E-10	−2.45	Down

### Gene Ontology analysis

**FIGURE 2:**
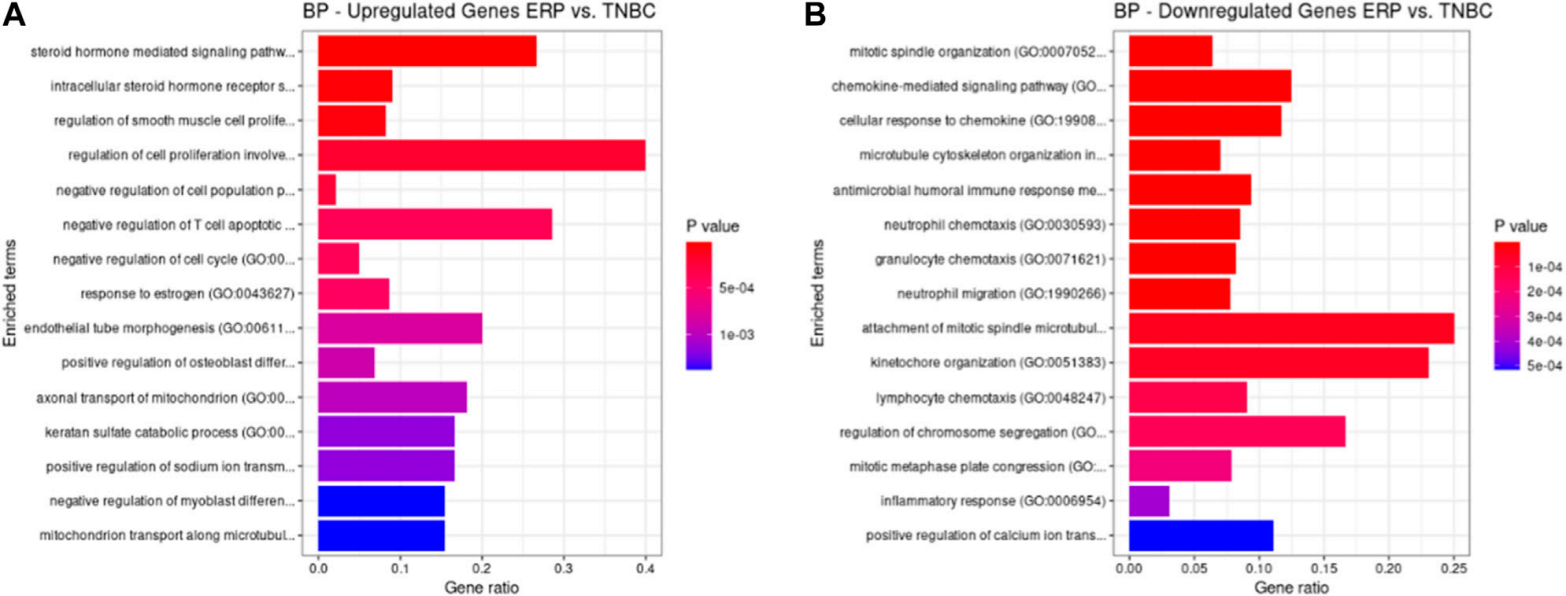
GO biological processes up- and downregulated by DEGs. Bar chart plots of top 15 BPs in ERP vs. TNBC. The *x*-axis is the gene ratio, while the color represents *p*-value. **(A)** Steroid hormone-mediated signaling pathway and response to estrogen and endothelial tube morphogenesis as significant upregulated BP. **(B)** Chemokine-mediated signaling pathway, cellular response to chemokine, granulocyte chemotaxis, and attachment of mitotic spindle microtubules to kinetochore as significant downregulated BP.

**FIGURE 3:**
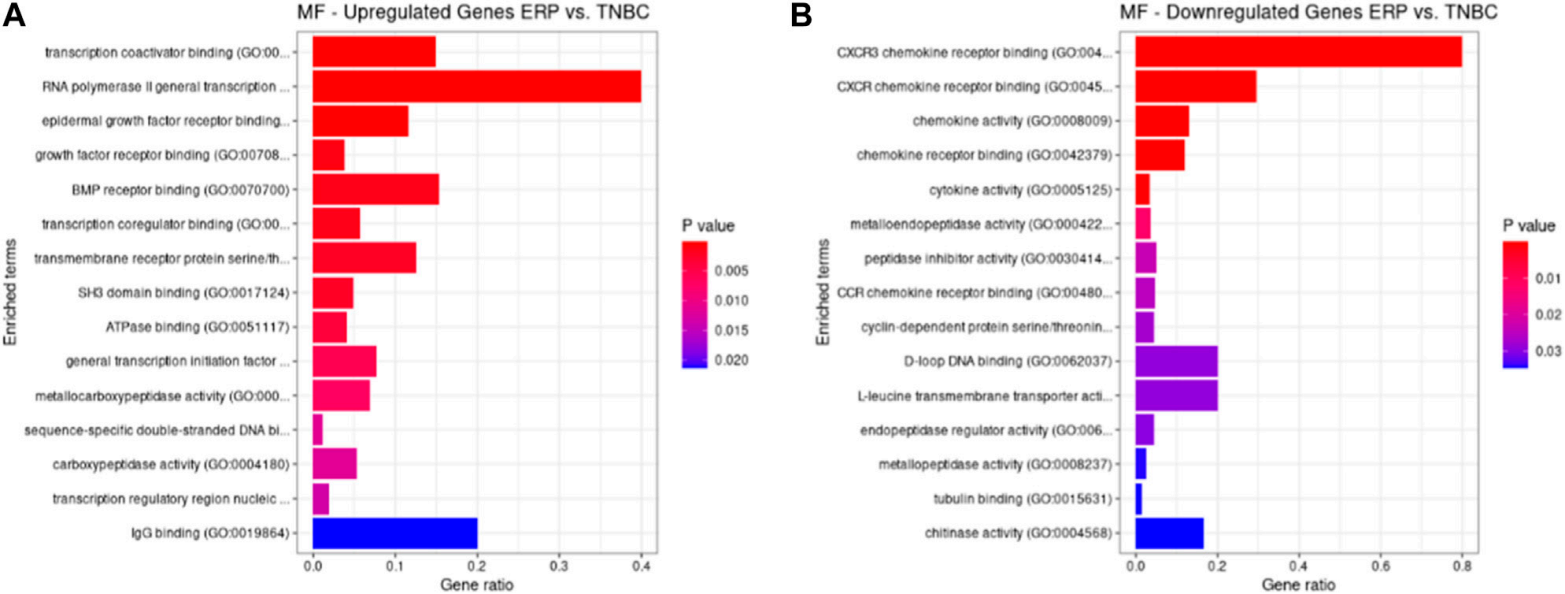
GO molecular functions up- and downregulated by DEGs. Bar chart plots of top 15 MF in ERP vs. TNBC. *X*-axis is the gene ratio, while the color represents *p*-value. **(A)** Transcription coactivator binding, RNA polymerase II general transcription initiation factor binding, epidermal growth factor receptor binding, and BMP receptor binding as significant upregulated MF. **(B)** CXCR3 chemokine receptor binding, CXCR chemokine receptor binding, chemokine activity, and L-leucine transmembrane transporter activity as significant downregulated MF.

**FIGURE 4:**
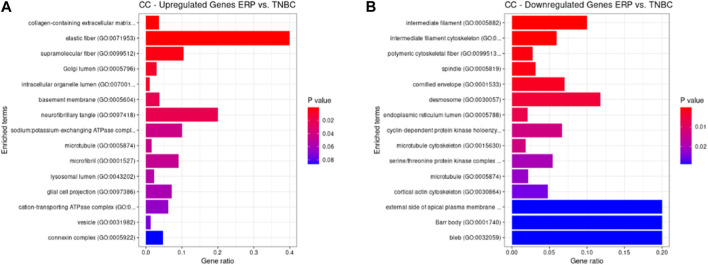
GO cellular components up- and downregulated by DEGs. Bar chart plots of top-15 CC in ERP vs. TNBC. *X*-axis is the gene ratio, while the color represents *p*-value. **(A)** Collagen-containing extracellular matrix, elastic fiber, Golgi lumen, and intracellular organelle lumen as significant upregulated CC. **(B)** Intermediate filament, intermediate filament cytoskeleton, polymeric cytoskeletal fiber, spindle, and desmosome as significant downregulated CC.

Both up- and downregulated DEGs were subjected to GO enrichment analysis. This analysis revealed BP, CC, and MF that were affected due to change in gene expression. Upregulated DEGs of biological processes were enriched in steroid hormone-mediated signaling pathway, intracellular steroid hormone receptor signaling pathway, regulation of smooth muscle cell proliferation, and response to estrogen, indicating that upregulated genes are involved in the regulation of breast stem cells, increased cell proliferation, increased estrogen hormone and cancerous T-cells, angiogenesis, and excessive mitochondrial and sodium ion transport (Figure 2A; Table 3). The downregulated DEGs enriched in mitotic spindle organization, chemokine-mediated signaling pathway, cellular response to chemokine, microtubule cytoskeleton organization involved in mitosis, antimicrobial humoral immune response mediated by antimicrobial peptides, neutrophil chemotaxis, granulocyte chemotaxis, and the attachment of mitotic spindle microtubules to kinetochore and kinetochore organization indicated that they may have cancer development-related functions because of disrupted cell signaling and a dysregulated cell cycle due to incorrectly organized proteins and a suppressed immune system (Figure 2B; Table 4). Alternatively, the upregulated DEGs of molecular functioning show transcription coactivator binding, RNA polymerase II general transcription initiation factor binding, epidermal growth factor receptor binding, BMP receptor binding, SH3 domain binding, ATPase binding, general transcription initiation factor binding, metallocarboxypeptidase activity, and IgG binding (Figure 3A; Table 5). This denotes disrupted cell signaling, increased cell proliferation, growth, differentiation, and epithelial–mesenchymal transition (EMT) due to upregulated transcription. On the other hand, the downregulated DEGs were mainly enriched in CXCR3 and CXCR chemokine receptor binding, chemokine and cytokine activity, peptidase inhibitor activity, L-leucine transmembrane transporter activity, and chitinase activity, which indicate suppressed immune system response and increased abnormal proteins which may result in cancer progression and development (Figure 3B; Table 6). The cellular component enrichment of upregulated DEGs, collagen-containing extracellular matrix, elastic fiber, Golgi lumen, intracellular organelle lumen, basement membrane, and sodium:potassium-exchanging ATPase complex indicate that these may have been involved in the initiation and progression of cancer due to changes in the cellular cytoskeleton, membrane remodeling, and alterations in protein secretions (Figure 4A; Table 7). Moreover, the downregulated DEGs of cellular components show intermediate filament, intermediate filament cytoskeleton, polymeric cytoskeletal fiber, spindle, cornified envelope, desmosome, and endoplasmic reticulum lumen, which indicate cancer development and progression due to disrupted cytoskeletal proteins, dysregulated cell division, misfolded proteins, and DNA damage (Figure 4B; Table 8).

**TABLE 3:** GO analysis of BP of upregulated DEGs according to Enrichr (*p*-value<0.05).

Biological process	Gene ratio	*p*-value	Genes
Steroid hormone-mediated signaling pathway	4/15	4.19E-07	BMP4; AR; PGR; ESR1
Intracellular steroid hormone receptor signaling pathway	4/44	3.79E-05	AR; SCGB2A1; PGR; ESR1
Regulation of smooth muscle cell proliferation	4/49	5.82E-05	BMP4; ELN; OGN; APOD
Regulation of cell proliferation involved in heart morphogenesis	2/5	0.00018	BMP4; TBX3
Negative regulation of cell population proliferation	8/379	0.00022	BMP4; AR; BTG2; CAMK2N1; ERBB4; OGN; APOD; TBX3
Negative regulation of cell cycle	4/80	0.00039	BMP4; BTG2; CAMK2N1; RHOB
Response to estrogen	3/35	0.00045	GSTM3; AR; ESR1
Endothelial tube morphogenesis	2/10	0.00080	BMP4; RHOB
Positive regulation of osteoblast differentiation	3/44	0.00089	BMP4; NPNT; IL6ST
Axonal transport of mitochondrion	2/11	0.00098	MAPT; SYBU
Keratan sulfate catabolic process	2/12	0.0011	OMD; OGN
Positive regulation of sodium ion transmembrane transport	2/12	0.0011	FXYD1; WNK4
Negative regulation of myoblast differentiation	2/13	0.0013	BMP4; TBX3
Mitochondrion transport along microtubule	2/13	0.0013	MAPT; SYBU

**TABLE 4:** GO analysis of BP of downregulated DEGs according to Enrichr (*p*-value<0.05).

Biological process	Gene ratio	*p*-value	Genes
Mitotic spindle organization	10/157	2.89E-08	CDC20; STMN1; NUF2; CDCA8; BIRC5; KIF23; KIF2C; BUB1; NDC80; AURKB
Chemokine-mediated signaling pathway	7/56	3.58E-08	CXCL10; CXCL9; FOXC1; CXCL11; CXCL8; CXCL13; CCL18
Cellular response to chemokine	7/60	5.85E-08	CXCL10; CXCL9; FOXC1; CXCL11; CXCL8; CXCL13; CCL18
Microtubule cytoskeleton organization involved in mitosis	9/128	6.26E-08	CDC20; STMN1; NUF2; CDCA8; BIRC5; KIF2C; BUB1; NDC80; AURKB
Antimicrobial humoral immune response mediated by antimicrobial peptide	6/64	2.00E-06	CXCL10; CXCL9; CXCL11; CXCL8; CXCL13; KRT6A
Neutrophil chemotaxis	6/70	3.40E-06	CXCL10; CXCL9; CXCL11; CXCL8; CXCL13; CCL18
Granulocyte chemotaxis	6/73	4.36E-06	CXCL10; CXCL9; CXCL11; CXCL8; CXCL13; CCL18
Neutrophil migration	6/77	5.95E-06	CXCL10; CXCL9; CXCL11; CXCL8; CXCL13; CCL18
Attachment of mitotic spindle microtubules to kinetochore	3/12	4.13E-05	NUF2; KIF2C; NDC80
Kinetochore organization	3/13	5.35E-05	CENPW; NUF2; NDC80
Lymphocyte chemotaxis	4/44	0.00012	CXCL10; CXCL11; CXCL13; CCL18
Regulation of chromosome segregation	3/18	0.00014	KIF2C; BUB1; AURKB
Mitotic metaphase plate congression	4/51	0.00022	NUF2; CDCA8; KIF2C; NDC80
Inflammatory response	7/230	0.00041	CXCL10; CXCL11; CXCL9; CXCL8; KRT16; CXCL13; CCL18
Positive regulation of calcium ion transmembrane transport	3/27	0.00051	CXCL10; CXCL9; CXCL11

**TABLE 5:** GO analysis of MF of upregulated DEGs according to Enrichr (*p*-value<0.05).

Molecular function	Gene ratio	*p*-value	Genes
Transcription coactivator binding	3/20	8.30E-05	AR; PGR; ESR1
RNA polymerase II general transcription initiation factor binding	2/5	0.00018	AR; ESR1
Epidermal growth factor receptor binding	3/26	0.00018	ERBB4; AGR2; CBLC
Growth factor receptor binding	4/105	0.0010	ERBB4; AGR2; CBLC; IL6ST
BMP receptor binding	2/13	0.0013	BMP4; GDF15
Transcription coregulator binding	3/53	0.0015	AR; PGR; ESR1
Transmembrane receptor protein serine/threonine kinase binding	2/16	0.0021	BMP4; GDF15
SH3 domain binding	3/62	0.0024	CBLC; EVL; MAPT
ATPase binding	3/73	0.0038	AR; PGR; ESR1
General transcription initiation factor binding	2/26	0.0055	AR; ESR1
Metallocarboxypeptidase activity	2/29	0.0068	CPA3; CPE
Sequence-specific double-stranded DNA binding	8/712	0.0114	FOXA1; AR; ERBB4; FOSB; FOS; LMX1B; ESR1; TBX3
Carboxypeptidase activity	2/38	0.0116	CPA3; CPE
Transcription regulatory region nucleic acid binding	4/212	0.0132	FOXA1; AR; ERBB4; FOS
IgG binding	1/5	0.0213	PIP

**TABLE 6:** GO analysis of MF of downregulated DEGs according to Enrichr (*p*-value<0.05).

Molecular function	Gene ratio	*p*-value	Genes
CXCR3 chemokine receptor binding	4/5	5.54E-09	CXCL10; CXCL11; CXCL9; CXCL13
CXCR chemokine receptor binding	5/17	3.68E-08	CXCL10; CXCL9; CXCL11; CXCL8; CXCL13
Chemokine activity	6/46	2.73E-07	CXCL10; CXCL9; CXCL11; CXCL8; CXCL13; CCL18
Chemokine receptor binding (GO:0042379)	6/50	4.54E-07	CXCL10; CXCL9; CXCL11; CXCL8; CXCL13; CCL18
Cytokine activity	6/173	0.00054	CXCL10; CXCL11; CXCL9; CXCL8; CXCL13; CCL18
Metalloendopeptidase activity	3/82	0.01235	ADAMDEC1; MMP7; MMP1
Peptidase inhibitor activity	2/40	0.02289	A2ML1; PI3
CCR chemokine receptor binding	2/42	0.02508	CXCL13; CCL18
Cyclin-dependent protein serine/threonine kinase regulator activity	2/44	0.02735	CCNB2; CDKN2A
D-loop DNA binding	1/5	0.02891	RAD51AP1
L-Leucine transmembrane transporter activity	1/5	0.02891	SLC7A5
Endopeptidase regulator activity	2/46	0.02970	A2ML1; PI3
Metallopeptidase activity	3/121	0.03415	ADAMDEC1; MMP7; MMP1
Tubulin binding	5/307	0.03443	STMN1; BIRC5; KIF23; KIF2C; FAM83D
Chitinase activity	1/6	0.03459	CHI3L2

**TABLE 7:** GO analysis of CC of upregulated DEGs according to Enrichr (*p*-value<0.05).

Cellular component	Gene ratio	*p*-value	s
Collagen-containing extracellular matrix	14/380	8.27E-10	CPA3; TPSB2; COL14A1; GDF15; ELN; HTRA1; NPNT; ASPN; THSD4; THBS4; MFAP4; CILP; OGN; COL4A5
Elastic fiber	2/5	0.00018	MFAP4; ELN
Supramolecular fiber	2/19	0.00298	MFAP4; ELN
Golgi lumen	3/100	0.00919	MUCL1; OMD; OGN
Intracellular organelle lumen	9/848	0.01058	BMP4; MUCL1; COL14A1; ERBB4; OMD; OGN; COL4A5; ABAT; HMGCS2
Basement membrane	2/52	0.02108	COL4A5; THBS4
Neurofibrillary tangle	1/5	0.02131	MAPT
Sodium:potassium-exchanging ATPase complex	1/10	0.04218	FXYD1
Microtubule	3/182	0.04380	KIF12; MAPT; SYBU
Microfibril	1/11	0.04630	MFAP4
Lysosomal lumen	2/86	0.05294	OMD; OGN
Glial cell projection	1/14	0.05856	MAPT
Cation-transporting ATPase complex	1/16	0.06664	FXYD1
Vesicle	3/226	0.07374	OGN; TSPAN1; SYBU
Connexin complex	1/21	0.08656	GJC3

**TABLE 8:** GO analysis of CC of downregulated DEGs according to Enrichr (*p*-value<0.05).

Cellular component	Gene ratio	*p*-value	Genes
Intermediate filament	5/50	1.08E-05	SYNM; KRT16; PKP1; KRT75; KRT6C
Intermediate filament cytoskeleton	5/84	0.00013	SYNM; KRT16; PKP1; KRT75; KRT6C
Polymeric cytoskeletal fiber	7/256	0.00077	SYNM; KRT16; PKP1; KIF23; KIF2C; KRT75; KRT6C
Spindle	6/192	0.00093	CDC20; BIRC5; KIF23; KIF2C; FAM83D; AURKB
Cornified envelope	3/43	0.00203	PKP1; PI3; DSC3
Desmosome	2/17	0.00435	PKP1; DSC3
Endoplasmic reticulum lumen	6/285	0.00664	SPP1; COL9A3; MELTF; MSLN; MFGE8; CP
Cyclin-dependent protein kinase holoenzyme complex	2/30	0.01326	CCNB2; CDK6
Microtubule cytoskeleton	6/331	0.01326	CDC20; CCNB2; KIF23; KIF2C; FAM83D; AURKB
Serine/threonine protein kinase complex	2/37	0.01977	CCNB2; CDK6
Microtubule	4/182	0.02221	BIRC5; KIF23; KIF2C; AURKB
Cortical actin cytoskeleton	2/42	0.02508	GYS2; SLC2A1
External side of apical plasma membrane	1/5	0.02891	SLC7A5
Barr body	1/5	0.02891	MACROH2A2
Bleb	1/5	0.02891	ANLN

### KEGG pathway analysis

**FIGURE 5:**
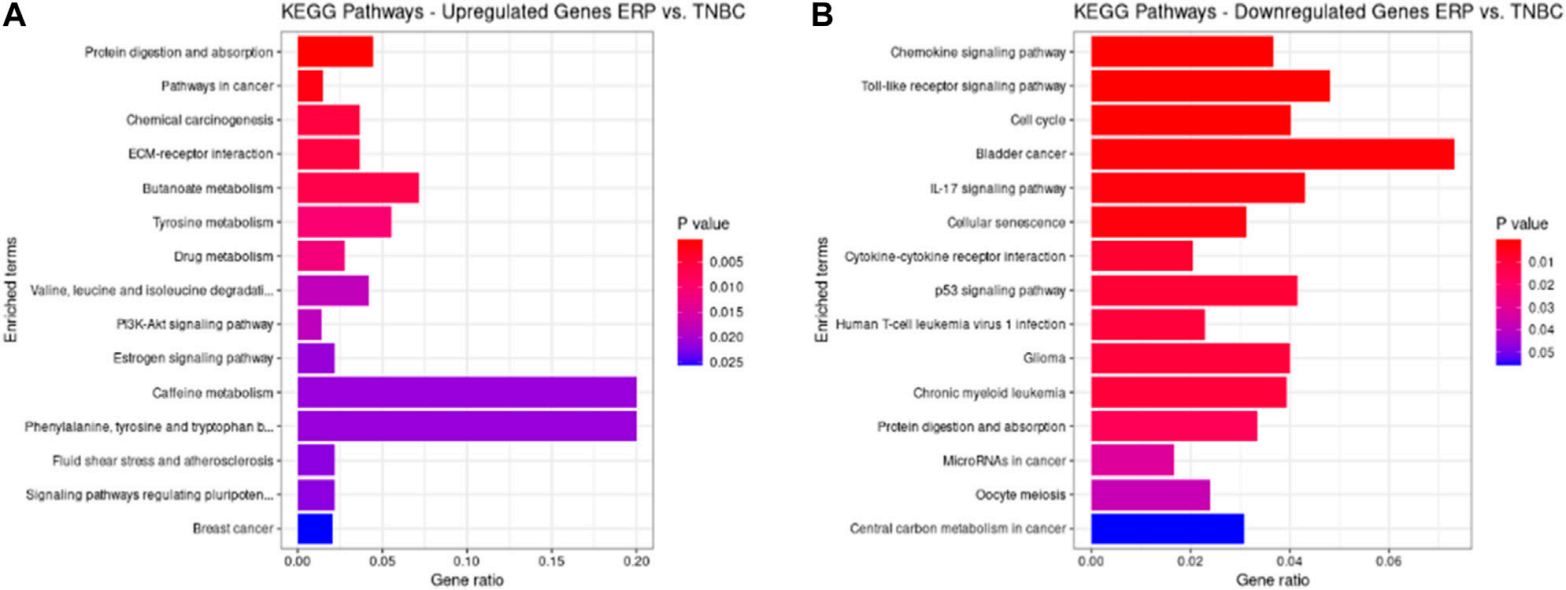
KEGG pathways analysis of upregulated DEGs. Bar chart plots of top 15 KEGG pathways in ERP vs. TNBC. *X*-axis is the gene ratio, while the color represents *p*-value. **(A)** Pathways in cancer, chemical carcinogenesis, ECM–receptor interaction, and tyrosine metabolism as significant upregulated pathways. **(B)** Chemokine signaling pathway, toll-like receptor signaling pathway, cell cycle, bladder cancer, and IL-17 signaling pathway as significant downregulated pathways.

KEGG pathways were predicted using Enrichr for the DEGs to identify biological pathways that are disrupted due to the up- and downregulation of genes involved in those pathways. As indicated in Figure 5A, upregulated genes such as BMP4, GSTM3, FOS, HMGCS2, ADH1B, COL4A5, ESR1, NAT1, IL6ST, and PGR were enriched in pathways in cancer, chemical carcinogenesis, ECM–receptor interaction, tyrosine metabolism, valine, leucine and isoleucine degradation, the PI3K-Akt signaling pathway, estrogen signaling pathway, caffeine metabolism, signaling pathways that regulate the pluripotency of stem cells, and BC (Table 9). This indicates that the upregulation of genes promotes pathways that are mainly involved in DNA repair, cell motility and proliferation, cell cycle regulation, the inhibition of apoptosis, and increased EMT, resulting in tumor development and prognosis. The downregulated genes such as CXCL8, CXCL10, CXCL11, CDC20, CDK6, CDKN2A, CXCL13, and SHC4 (Table 10) were enriched in chemokine signaling pathway, toll-like receptor signaling pathway, cell cycle, bladder cancer, IL-17 signaling pathway, cellular senescence, p53 signaling pathway, and microRNAs in cancer (Figure 5B). The downregulation of genes involved in these pathways plays a crucial role in the tumor microenvironment by disrupting immune response, cell cycle arrest in the G2/M phase, increased cell growth, metastasis, proliferation and invasiveness, and the angiogenic potential of cancer cells. The analysis revealed that cancer-related pathways that were dysregulated due to DEGs have also been reported in various other cancers.

**TABLE 9:** Pathway prediction for upregulated DEGs according to Enrichr (*p*-value<0.05).

KEGG pathway	Gene ratio	*p*-value	Genes
Protein digestion and absorption	4/90	0.00061	CPA3; COL14A1; ELN; COL4A5
Pathways in cancer	8/530	0.00200	BMP4; AR; GSTM3; IGF2; COL4A5; FOS; IL6ST; ESR1
Chemical carcinogenesis	3/82	0.00532	GSTM3; NAT1; ADH1B
ECM–receptor interaction	3/82	0.00532	CHAD; COL4A5; THBS4
Butanoate metabolism	2/28	0.00642	ABAT; HMGCS2
Tyrosine metabolism	2/36	0.01047	ADH1B; TAT
Drug metabolism	3/108	0.01133	GSTM3; NAT1; ADH1B
Valine, leucine, and isoleucine degradation	2/48	0.01813	ABAT; HMGCS2
PI3K–Akt signaling pathway	5/354	0.01831	ERBB4; CHAD; IGF2; COL4A5; THBS4
Estrogen signaling pathway	3/137	0.02129	PGR; FOS; ESR1
Caffeine metabolism	1/5	0.02131	NAT1
Phenylalanine, tyrosine, and tryptophan biosynthesis	1/5	0.02131	TAT
Fluid shear stress and atherosclerosis	3/139	0.02211	BMP4; GSTM3; FOS
Signaling pathways regulating pluripotency of stem cells	3/139	0.02211	BMP4; IL6ST; TBX3
BC	3/147	0.02555	PGR; FOS; ESR1

**TABLE 10:** Pathway prediction for downregulated DEGs according to Enrichr (*p*-value<0.05).

KEGG pathway	Gene ratio	*p*-value	Genes
Chemokine signaling pathway	7/190	0.00012	SHC4; CXCL10; CXCL11; CXCL9; CXCL8; CXCL13; CCL18
Toll-like receptor signaling pathway	5/104	0.00036	CXCL10; CXCL11; CXCL9; CXCL8; SPP1
Cell cycle	5/124	0.00081	CDC20; CCNB2; CDK6; CDKN2A; BUB1
Bladder cancer	3/41	0.00176	CXCL8; CDKN2A; MMP1
IL-17 signaling pathway	4/93	0.00217	CXCL10; CXCL8; MMP1; LCN2
Cellular senescence	5/160	0.00251	CCNB2; CDK6; CXCL8; CDKN2A; MYBL2
Cytokine–cytokine receptor interaction	6/294	0.00769	CXCL10; CXCL11; CXCL9; CXCL8; CXCL13; CCL18
p53 signaling pathway	3/72	0.00868	CCNB2; CDK6; CDKN2A
Human T-cell leukemia virus 1 infection	5/219	0.00936	CDC20; CCNB2; MMP7; CDKN2A; SLC2A1
Glioma	3/75	0.00970	SHC4; CDK6; CDKN2A
Chronic myeloid leukemia	3/76	0.01006	SHC4; CDK6; CDKN2A
Protein digestion and absorption	3/90	0.01585	KCNK5; COL9A3; KCNN4
MicroRNAs in cancer	5/299	0.03125	SHC4; CDK6; CDKN2A; STMN1; KIF23
Oocyte meiosis	3/125	0.03706	CDC20; CCNB2; BUB1
Central carbon metabolism in cancer	2/65	0.05559	SLC7A5; SLC2A1

### Selection of immune system genes

A total of 86 upregulated and 117 downregulated genes were used as a query against the marker genes database of the ScType R package, revealing that three upregulated and 10 downregulated genes were directly involved in immune system-related functions (Table 11, 12).

**TABLE 11:** Shortlisted upregulated DEGs involved in the immune system (*p*-value<0.05).

Gene name	*p*-value	LogFC	Function	Expression
CPA3	1.02E-05	1.84048	Generates mature protease; released by mast cells	Up
THBS4	1.60E-05	1.95670	Adhesive glycoprotein	Up
CXCL14	5.20E-05	2.25235	Chemotactic factor for monocytes	Up

**TABLE 12:** Shortlisted downregulated DEGs involved in the immune system (*p*-value<0.05).

Gene name	*p*-value	LogFC	Function	Expression
MELTF	1.66E-10	−1.85184	Cell surface glycoprotein	Down
STMN1	4.25E-08	−1.53543	Integrate intracellular regulatory signals	Down
CXCL8	5.51E-05	−1.97224	Chemotactic factor	Down
CXCL11	0.00020	−1.67806	Regulate cell trafficking	Down
PI3	0.00020	−1.59618	Antimicrobial peptide	Down
CXCL10	0.00022	−2.01352	Stimulates monocytes, natural killer, and T-cells migration	Down
CD24	0.27436	−1.86584	Essential role in cell differentiation	Down
CD24P4	0.28050	−1.83388	Pseudogene	Down
CCL18	0.00771	−1.73928	Chemotactic factor, attracts only lymphocytes	Down
CXCL13	0.01855	−1.62905	Chemotactic factor for B-lymphocytes	Down

### Isoform switching

Isoform switching analysis was performed on shortlisted immune system related genes, facilitating the identification of known and novel isoform switches from RNA-seq derived quantification data. Of 10 downregulated immune genes, three (STMN1, MELTF, and CXCL8) were found to have isoforms that were significantly used in ERP and have been validated through the Expression Atlas (Supplementary Information Table S3). Moreover, no significant isoform switch was observed in upregulated immune genes.

### Isoform usage

**FIGURE 6:**
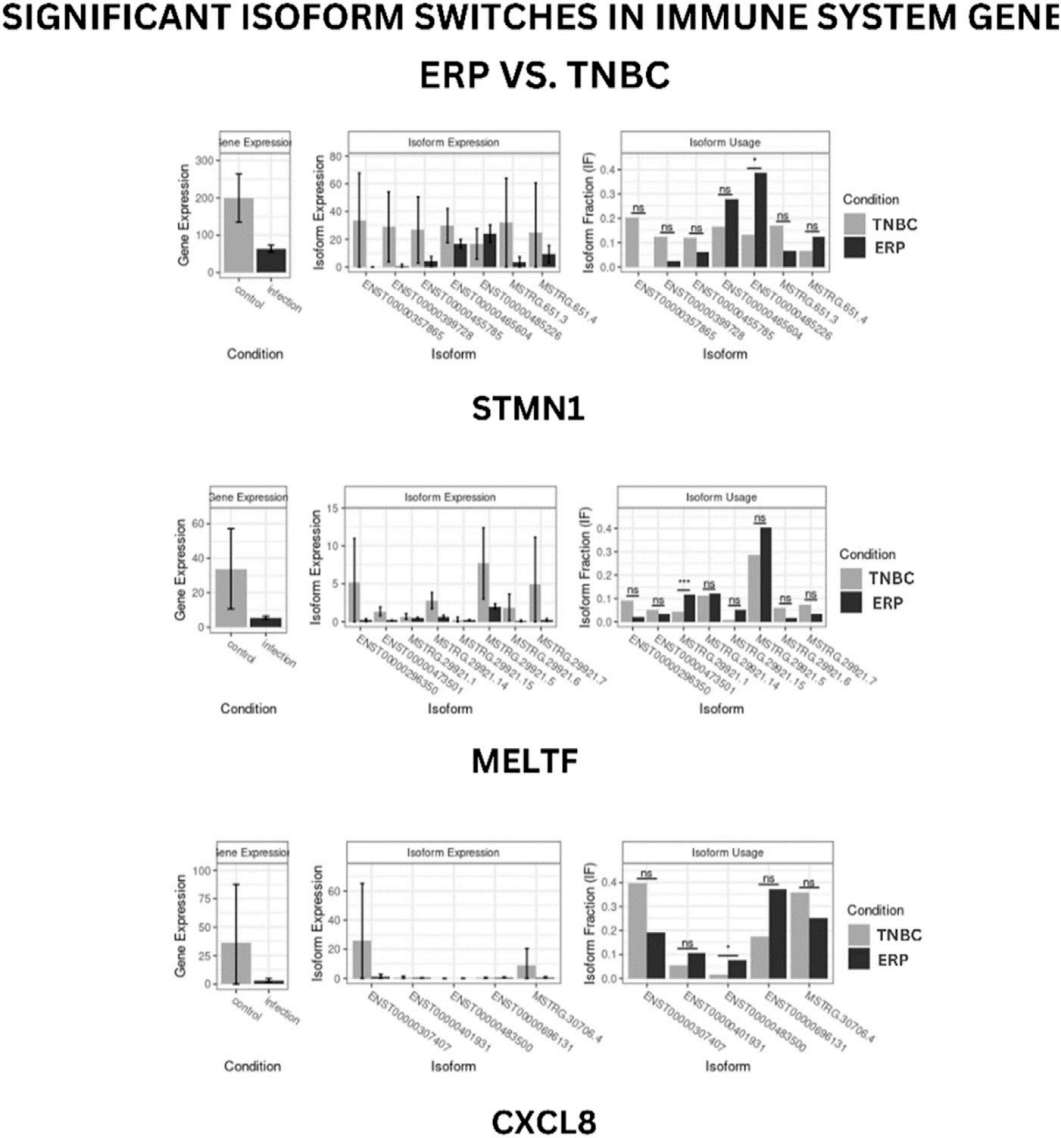
Isoform switch of differentially expressed immune genes. “*” represents significant isoform usage.

STMN1, MELTF, and CXCL8 represent significant switches in isoform usage across ERP vs. TNBC, as shown in sashimi plots in Supplementary Information Figures S1–S3 respectively. By comparing the isoform usage across conditions, it was revealed that STMN1 has a single isoform (ENST00000485226) which was overexpressed in ERP. CXCL8 also has one isoform (ENST00000483500) which was significantly used in ERP. Furthermore, it was found that a novel isoform (MSTRG.29921.1) of MELTF was overexpressed in ERP (Figure 6).

**FIGURE 7:**
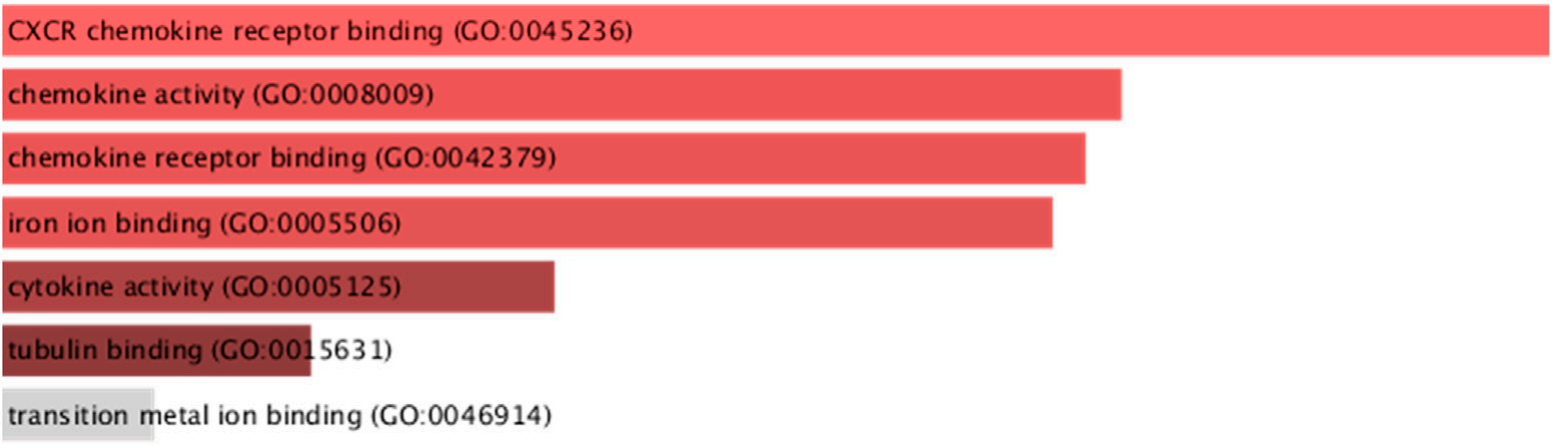
GO molecular functions of downregulated immune system genes (STMN1, MELTF, and CXCL8) having significant isoform switches (*p*-value <0.05).

Gene Ontology analysis of downregulated immune genes indicates that these genes may be involved in key immune system molecular functions such as CXCR chemokine receptor binding, chemokine activity, iron ion binding, and cytokine activity (Figure 7).

## Discussion

BC is the most prevalent type of cancer worldwide. It is thus essential to understand and explore ways to prevent its occurrence while identifying the genetic changes that are more susceptible to its incidence. The present study identified 9,771 DEGs, of which 86 genes were significantly upregulated and 117 were downregulated. The identified upregulated genes were FOXA1, RHOB, AR, CMBL, AGR2, ESR1, TFF3, SYBU, CBLC, and DNALI1; the downregulated genes were CENPW, EN1, A2ML1, TMSB15A, FOXC1, KRT16, SLC7A5, CDK6, MELTF, and CA9. Functional enrichment analysis of these DEGs revealed that the intracellular steroid hormone receptor signaling pathway, chemokine-mediated signaling pathway, kinetochore organization, pathways in cancer, BC, toll-like receptor signaling pathway, and cell cycle were the most dysregulated biological pathways and processes.

In the present study, FOXA1 was found to be upregulated and has been reported to inhibit STAT2, a transcription factor and its target IFN signaling pathway in BC; this may result in cancer progression due to suppressed immune response (He et al., 2021). Furthermore, upregulated RHOB results in ER-α (estrogen receptor alpha) overexpression that leads to increased estrogen uptake by BC cells which helps them grow and proliferate (Médale-Giamarchi et al., 2013). It has been reported that AR overexpression increases the transcription of genes involved in the cell cycle, resulting in increased proliferation of prostate cancer cells (Formaggio et al., 2021). This study found that CMBL (*p*-value: 0.00003) is suppressed in TNBC compared to non-TNBC types of BC, such as ERP. It encodes a cysteine hydrolase that cleaves cyclic esters which activate an angiotensin receptor blocker that helps lower blood pressure (Guo et al., 2017). Upregulated AGR2 is found in BC due to ER signaling and endoplasmic reticulum stress, and it results in increased cell proliferation, survival, and metastasis in BC (Ann et al., 2018). Moreover, ESR1 upregulation makes BC cells more prone to estrogen uptake which may lead to the increased growth and proliferation of cancer cells (Lei et al., 2019). According to the literature, TFF3 acts as an oncogene because it regulates other genes (FOXA1, HER2, and AR) involved in EMT, thus promoting invasiveness, survival, and increased proliferation in multiple carcinomas such as gastric cancer, mammary carcinoma, and prostate cancer (Yuan et al., 2017). It has been reported that SYBU, a microtubule-associated protein, is overexpressed in hepatocellular carcinoma (HCC), which results in disrupted cell cycle and increased proliferation (Zheng and Yu, 2021). Breast tumor formation is increased by CBLC overexpression, which suppresses TGF-β (transforming growth factor beta). This results in the deactivation of its target Smad3 pathway which is responsible for proliferation, differentiation, and apoptosis (Kang et al., 2012). This study found that DNALI1 (*p*-value: 0.0000148), a flagellar protein, is overexpressed in BC, which has not been reported previously for any other carcinoma.

According to the literature, CENPW was downregulated in BC and HCC. It is involved in kinetochore organization and centromere complex assembly. This downregulation results in subsequent function disruption, resulting in chromosomal instability due to mis-segregation of chromosomes (Liu and Liu, 2022). It has been reported that EN1 is downregulated in lung cancer due to altered DNA methylation which promotes cell proliferation and differentiation (Jiang et al., 2017). The downregulation of A2ML1, a protease inhibitor, results in MAPK pathway mutation, which leads to apoptotic resistance and uncontrolled cell division in BC (Li et al., 2016). FOXC1 suppression induces ER-α expression in BC cells, which helps in increased estrogen uptake, resulting in the growth and proliferation of tumor cells (Wang et al., 2017). It has been reported that KRT16 is overexpressed in basal-like TNBC, along with increased expression of EMT-associated proteins. In contrast, in luminal A and B subtypes of BC which include ER^+^ and PR^+^ tumors, KRT16 expression was suppressed, but E-cadherin (CDH1), an EMT protein was overexpressed, leading to metastasis due to increased cellular motility (Elazezy et al., 2021). SLC7A5 has been reported to be overexpressed in TNBC due to its glutamine transporting activity to tumor cells for energy production—TNBC is thus glutamine dependent and requires glutaminase for its catabolism. In addition, cells with increased proliferation use transaminases to catabolize glutamate, in contrast to glutamate dehydrogenase (GLUD), to reduce ammonia production. However, ER^+^ tumors are glutamine-independent and show increased GLUD expression (Wang et al., 2020). It has been reported that the downregulation of CDK6 also suppresses its interacting gene, RB1—a tumor suppressor gene. This results in dysregulated cell growth, apoptosis, and increased proliferation in tumor cells (Knudsen et al., 2020). MELTF downregulation also dysregulates its interacting genes such as ACO2, a gene-encoding Krebs’ cycle enzyme. The disruption of Krebs’ cycle enzymes leads to the production of oncometabolites, which stabilize hypoxia-inducible factor 1 and activate cell growth signaling by regulating DNA methylation—crucial factors in cancer progression (Sajnani et al., 2017). CA9 suppression leads to the disruption of interacting genes such as HIF3A and EPAS1 which are involved in regulating hypoxic conditions. Such conditions are favorable for the increased proliferation of tumor cells (Jun et al., 2017). TMSB15A has been reported to be upregulated in TNBC; it plays a crucial role in the organization of the cytoskeleton, which is responsible for cancer cell motility and is involved in cancer metastasis (Darb-Esfahani et al., 2012).

KEGG pathway enrichment analysis revealed protein digestion and absorption, pathways in cancer, chemical carcinogenesis, and ECM–receptor interaction as upregulated pathways, while downregulated pathways include chemokine signaling, toll-like receptor signaling, cell cycle, bladder cancer, IL-17 signaling, cellular senescence, cytokine–cytokine receptor interaction, and p53 signaling. We found that the protein digestion and absorption pathway was upregulated, which demonstrates the use of proteins as an alternative fuel by tumor cells to fulfill their metabolic needs. This occurs due to the limited supply and metabolism of glucose by cancer cells (Lieu et al., 2020). Moreover, it was found that pathways in cancer were upregulated that involve the disruption of the ErbB, p-53-mediated apoptotic, and GSK3 signaling pathways, which are involved in DNA repair, cell growth, migration, differentiation, and metabolism (Yip and Papa, 2021). The upregulation of chemical carcinogenesis activates certain hormonal pathways that make mammary glands more susceptible to carcinogenesis due to altered DNA repair genes (Rodgers et al., 2018). Furthermore, upregulated ECM–receptor interaction results in interaction with HMMR and SDC1 genes, the dysregulation of which promotes BC cell motility and differentiation (Yeh et al., 2018). A downregulated chemokine signaling pathway and cytokine–cytokine receptor interaction cannot recruit immune cells (leukocytes) to the tumor microenvironment, thus resulting in tumor progression (Gil Del Alcazar et al., 2020). The downregulation of toll-like receptor signaling pathways results in non-recognition and the escape of cancer cells from the immune system, leading to the invasiveness, migration, and angiogenic potential of cancer cells (Javaid and Choi, 2020). The disruption of the cell cycle at the G2/M phase results in cells that contain damaged DNA and genomic instability, a hallmark of cancer (Thu et al., 2018). It has been reported that increased ER-α in BC cells suppresses bladder cancer cell growth by downregulating INPP4B which, in turn, suppresses the AKT signaling pathway (Hsu et al., 2014). Research has found that the IL-17 signaling pathway becomes downregulated due to increased estrogen receptor expression, resulting in dysregulated PD-1/PD-L1 and CD8^+^ T cell expression—a suppressed immune response (Shuai et al., 2020). Furthermore, downregulated cellular senescence results in increased cell proliferation and tumor development (Milczarek, 2020). Moreover, a downregulated p53 signaling pathway cannot perform DNA damage repair and cell death, thereby facilitating the increased growth and metastasis of tumor cells (Marei et al., 2021).

According to the GTEx portal, cells express an average of 3.42 transcripts per gene (Tung et al., 2020). The expression dominance of major isoform transcripts compared to others from the same gene is crucial for normal cellular homeostasis (Hu et al., 2017). However, splicing regulation is often disrupted in cancer with a dominant expression of alternative transcripts in a tumor microenvironment (TME) which promotes switches that contribute to tumor progression and metastasis (Kahraman et al., 2020). The interaction pattern of the cancer-specific most dominant transcript (cMDT) differs from the generally expressed isoform of normal cells because of changes caused by alternative splicing; this could affect protein domains due to mutations caused by tumor and subsequent disruption in cancer-related pathways (Yang et al., 2016; Climente-González et al., 2017). According to the literature, apoptosis, ubiquitin, signaling, and spliceosomes were the most disrupted protein interactions (Kahraman et al., 2020). It has been reported that isoform switching leads to the loss of the DNA sequence that encodes for protein domains, promoting functional loss. The subsequent switches have functional consequences for cancer development and progression (Vitting-Seerup and Sandelin, 2017).

The present study has identified the differential usage of transcript isoforms among ERP and TNBC. It revealed isoforms that are significantly expressed and used by shortlisted downregulated genes (STMN1, MELTF, and CXCL8). CXCL8 encodes a protein that is involved in chemotaxis (Łukaszewicz-Zając et al., 2020). It transcribes five transcripts; among them, only one isoform transcript was significantly used. Differential gene expression plotting shows that CXCL8 is downregulated in ERP (Figure 6). On the other hand, there is in isoform usage an increased use of isoform ENST00000483500 in ERP. However, this isoform is non-coding due to retained introns and the unavailability of any domain, leading to functional loss of CXCL8 and immune suppression as a consequence of the non-recruitment of macrophages and neutrophils to the TME (Xiong et al., 2022). STMN1, a cytosolic phosphoprotein, is involved in microtubule destabilization by regulating the microtubule filament system and signal transduction (Bao et al., 2017). It transcribes four known and two novel isoforms (Figure 6). The isoform usage plot indicates that a single isoform (ENST00000485226) is significantly used in ERP, as compared to TNBC. However, it lacks a domain, which results in its non-coding behavior. Furthermore, STMN1 is repressed in ERP, as shown in differential gene expression plotting which promotes ERP progression due to the disruption of microtubules and their subsequent role in the growth of immune cells such as T-cells and natural killer cells (Zhang et al., 2022). MELTF, a cell surface glycoprotein, is involved in cellular iron uptake (Sawaki et al., 2019). MELTF could transcribe six novel and two known isoforms (Figure 6); however, increased use of the single isoform MSTRG.29921.1 has been identified in ERP. Moreover, this isoform contains nonsense codons that prematurely terminate translation—nonsense-mediated decay (NMD). Differential gene expression plotting also shows that MELTF is downregulated in ERP, leading to the decreased proliferation and maturation of immune cells such as lymphocytes due to decreased iron uptake (Roemhild et al., 2021). Gene Ontology analysis of shortlisted immune genes revealed that CXCR chemokine receptor binding, iron ion binding, and cytokine activity are the most dysregulated molecular functions (Figure 7). These functions mediate immune response by recruiting immune cells such as monocytes, T cells, lymphocytes, and natural killer cells, and assist their growth and proliferation. The downregulation of these functions promotes ERP BC due to suppressed immune response in TME (Bates et al., 2018).

This research therefore provides key insights into the genes that are differentially expressed in ERP. Moreover, DNALI1 is a novel gene that has not been previously reported and is involved in ERP BC development. Furthermore, the identification of three immune system-related genes (STMN1, MELTF, and CXCL8) reveals that the dysregulation of the immune system due to isoform switching is the major factor in ERP BC development and progression. Downregulation and isoform switching of key immune system genes suggest BC progression and possible metastasis due to the non-recruitment of cytokines in the TME.

## Conclusion

ERP BC is characterized by the growth of tumor cells in response to estrogen hormone. The dysregulation of gene expression results in the development of significant biological changes that are key features of multiple human carcinomas such as prostate cancer, gastric cancer, hepatocellular carcinoma, and lung cancer. In this study, 9,771 DEGs were identified; among these, 86 genes were upregulated and 117 were downregulated. Six genes (FOXA1, RHOB, AGR2, ESR1, CBLC, and FOXC1) were found to be significantly associated with the development and progression of ERP BC. This study also identified a novel set of genes (DNALI1, TMSB15A, AR, TFF3, SYBU, CENPW, EN1, CDK6, MELTF, and CA9) not previously reported positive for estrogen receptors but that has been reported in other carcinomas. Moreover, alternative splicing and subsequent isoform expression in three downregulated immune system genes (STMN1, MELTF, and CXCL8) had been identified that were mainly responsible for ERP progression due to suppression of the immune system and the non-recruitment of cytokines against cancer cells. It was found that CXCR chemokine receptor binding, iron ion binding, and cytokine activity were the most dysregulated functions due to immune system suppression. This study reveals that dysregulation of the immune system due to isoform switching is the major factor in ERP BC development and progression. Therefore, these crucial immune system genes should be targeted as therapeutic biomarkers.

## Data Availability

The original contributions presented in the study are included in the article/Supplementary Material, further inquiries can be directed to the corresponding author.

## References

[B1] Ann P. Seagle B.-L. L. Shilpi A. Kandpal M. Shahabi S. (2018). Association of increased primary breast tumor AGR2 with decreased disease-specific survival. Oncotarget 9, 23114–23125. 10.18632/oncotarget.25225 29796176 PMC5955412

[B2] Bao P. Yokobori T. Altan B. Iijima M. Azuma Y. Onozato R. (2017). High STMN1 expression is associated with cancer progression and chemo-resistance in lung squamous cell carcinoma. Ann. Surg. Oncol. 24, 4017–4024. 10.1245/s10434-017-6083-0 28933054

[B3] Baralle F. E. Giudice J. (2017). Alternative splicing as a regulator of development and tissue identity. Nat. Rev. Mol. Cell Biol. 18, 437–451. 10.1038/nrm.2017.27 28488700 PMC6839889

[B4] Bates J. P. Derakhshandeh R. Jones L. Webb T. J. (2018). Mechanisms of immune evasion in breast cancer. BMC cancer 18, 556–614. 10.1186/s12885-018-4441-3 29751789 PMC5948714

[B5] Chen S. Zhou Y. Chen Y. Gu J. (2018). fastp: an ultra-fast all-in-one FASTQ preprocessor. Bioinformatics 34, i884–i890. 10.1093/bioinformatics/bty560 30423086 PMC6129281

[B6] Chen Y.-L. Wang K. Xie F. Zhuo Z.-L. Liu C. Yang Y. (2022). Novel biomarkers identified in triple-negative breast cancer through RNA-sequencing. Clin. Chim. Acta 531, 302–308. 10.1016/j.cca.2022.04.990 35504321

[B7] Climente-González H. Porta-Pardo E. Godzik A. Eyras E. (2017). The functional impact of alternative splicing in cancer. Cell Rep. 20, 2215–2226. 10.1016/j.celrep.2017.08.012 28854369

[B8] Costa-Silva J. Domingues D. Lopes F. M. (2017). RNA-seq differential expression analysis: an extended review and a software tool. PloS one 12, e0190152. 10.1371/journal.pone.0190152 29267363 PMC5739479

[B9] Danecek P. Bonfield J. K. Liddle J. Marshall J. Ohan V. Pollard M. O. (2021). Twelve years of SAMtools and BCFtools. Gigascience 10, giab008. 10.1093/gigascience/giab008 33590861 PMC7931819

[B10] Darb-Esfahani S. Kronenwett R. Von Minckwitz G. Denkert C. Gehrmann M. Rody A. (2012). Thymosin beta 15A (TMSB15A) is a predictor of chemotherapy response in triple-negative breast cancer. Br. J. cancer 107, 1892–1900. 10.1038/bjc.2012.475 23079573 PMC3504944

[B11] Elazezy M. Schwentesius S. Stegat L. Wikman H. Werner S. Mansour W. Y. (2021). Emerging insights into keratin 16 expression during metastatic progression of breast cancer. Cancers 13, 3869. 10.3390/cancers13153869 34359774 PMC8345379

[B12] Fatima F. Saleem S. Hameed A. Haider G. Ali Zaidi S. A. Kanwal M. (2019). Association analysis and allelic distribution of deletion in CC chemokine receptor 5 gene (CCR5Δ32) among breast cancer patients of Pakistan. Mol. Biol. Rep. 46, 2387–2394. 10.1007/s11033-019-04699-6 30848448

[B13] Formaggio N. Rubin M. A. Theurillat J.-P. (2021). Loss and revival of androgen receptor signaling in advanced prostate cancer. Oncogene 40, 1205–1216. 10.1038/s41388-020-01598-0 33420371 PMC7892335

[B14] Frazee A. C. Pertea G. Jaffe A. E. Langmead B. Salzberg S. L. Leek J. T. (2015). Ballgown bridges the gap between transcriptome assembly and expression analysis. Nat. Biotechnol. 33, 243–246. 10.1038/nbt.3172 25748911 PMC4792117

[B15] Frazee A. C. Pertea G. Jaffe A. E. Langmead B. Salzberg S. L. Leek J. T. (2014). Flexible isoform-level differential expression analysis with Ballgown. Biorxiv, 003665. 10.1101/003665

[B16] Gil Del Alcazar C. R. Alečković M. Polyak K. (2020). Immune escape during breast tumor progression. Cancer Immunol. Res. 8, 422–427. 10.1158/2326-6066.CIR-19-0786 32238387 PMC7138346

[B17] Gonzalez H. Hagerling C. Werb Z. (2018). Roles of the immune system in cancer: from tumor initiation to metastatic progression. Genes and Dev. 32, 1267–1284. 10.1101/gad.314617.118 30275043 PMC6169832

[B18] Guo J. Gong G. Zhang B. (2017). Screening and identification of potential biomarkers in triple-negative breast cancer by integrated analysis. Oncol. Rep. 38, 2219–2228. 10.3892/or.2017.5911 28849078

[B19] He Y. Wang L. Wei T. Xiao Y.-T. Sheng H. Su H. (2021). FOXA1 overexpression suppresses interferon signaling and immune response in cancer. J. Clin. Investigation 131, e147025. 10.1172/JCI147025 PMC8279591 34101624

[B20] Hsu I. Yeh C.-R. Slavin S. Miyamoto H. Netto G. J. Muyan M. (2014). Estrogen receptor alpha prevents bladder cancer via INPP4B inhibited akt pathway *in vitro* and *in vivo* . Oncotarget 5, 7917–7935. 10.18632/oncotarget.1421 25277204 PMC4202170

[B21] Hu J. Boritz E. Wylie W. Douek D. C. (2017). Stochastic principles governing alternative splicing of RNA. PLoS Comput. Biol. 13, e1005761. 10.1371/journal.pcbi.1005761 28910283 PMC5614656

[B22] Ianevski A. Giri A. K. Aittokallio T. (2022). Fully-automated and ultra-fast cell-type identification using specific marker combinations from single-cell transcriptomic data. Nat. Commun. 13, 1246. 10.1038/s41467-022-28803-w 35273156 PMC8913782

[B23] Javaid N. Choi S. (2020). Toll-like receptors from the perspective of cancer treatment. Cancers 12, 297. 10.3390/cancers12020297 32012718 PMC7072551

[B24] Jiang C.-L. He S.-W. Zhang Y.-D. Duan H.-X. Huang T. Huang Y.-C. (2017). Air pollution and DNA methylation alterations in lung cancer: A systematic and comparative study. Oncotarget 8, 1369–1391. 10.18632/oncotarget.13622 27901495 PMC5352062

[B25] Jun J. C. Rathore A. Younas H. Gilkes D. Polotsky V. Y. (2017). Hypoxia-inducible factors and cancer. Curr. Sleep. Med. Rep. 3, 1–10. 10.1007/s40675-017-0062-7 28944164 PMC5607450

[B26] Kahraman A. Karakulak T. Szklarczyk D. Von Mering C. (2020). Pathogenic impact of transcript isoform switching in 1,209 cancer samples covering 27 cancer types using an isoform-specific interaction network. Sci. Rep. 10, 14453. 10.1038/s41598-020-71221-5 32879328 PMC7468103

[B27] Kang J. M. Park S. Kim S. J. Hong H. Jeong J. Kim H. (2012). CBL enhances breast tumor formation by inhibiting tumor suppressive activity of TGF-β signaling. Oncogene 31, 5123–5131. 10.1038/onc.2012.18 22310290

[B28] Kim D. Langmead B. Salzberg S. L. (2015). Hisat: A fast spliced aligner with low memory requirements. Nat. methods 12, 357–360. 10.1038/nmeth.3317 25751142 PMC4655817

[B29] Knudsen E. S. Nambiar R. Rosario S. R. Smiraglia D. J. Goodrich D. W. Witkiewicz A. K. (2020). Pan-cancer molecular analysis of the RB tumor suppressor pathway. Commun. Biol. 3, 158. 10.1038/s42003-020-0873-9 32242058 PMC7118159

[B30] Lamb C. A. Vanzulli S. Lanari C. L. M. (2019). Hormone receptors in breast cancer: more than estrogen receptors. Med. (B Aires) 79, 540–545. 31864223

[B31] Lei J. T. Gou X. Seker S. Ellis M. J. (2019). ESR1 alterations and metastasis in estrogen receptor positive breast cancer. J. cancer metastasis Treat. 5, 38. 10.20517/2394-4722.2019.12 31106278 PMC6519472

[B32] Li G. Hu J. Hu G. (2017). Biomarker studies in early detection and prognosis of breast cancer. Transl. Res. Breast Cancer Biomark. Diagnosis, Target. Ther. Approaches Precis. Med. 1026, 27–39. 10.1007/978-981-10-6020-5_2 29282678

[B33] Li Y. Tang X.-Q. Bai Z. Dai X. (2016). Exploring the intrinsic differences among breast tumor subtypes defined using immunohistochemistry markers based on the decision tree. Sci. Rep. 6, 35773. 10.1038/srep35773 27786176 PMC5082366

[B34] Lieu E. L. Nguyen T. Rhyne S. Kim J. (2020). Amino acids in cancer. Exp. Mol. Med. 52, 15–30. 10.1038/s12276-020-0375-3 31980738 PMC7000687

[B35] Liu X. Liu Y. (2022). Comprehensive analysis of the expression and prognostic significance of the CENP family in breast cancer. Int. J. General Med. 15, 3471–3482. 10.2147/IJGM.S354200 PMC8976518 35378917

[B36] Łukaszewicz-Zając M. Pączek S. Mroczko B. (2020). The significance of chemokine CXCL-8 in esophageal carcinoma. Archives Med. Sci. 13. 10.5114/aoms.2017.71933 PMC7069419 32190161

[B37] Marei H. E. Althani A. Afifi N. Hasan A. Caceci T. Pozzoli G. (2021). p53 signaling in cancer progression and therapy. Cancer Cell Int. 21, 703–715. 10.1186/s12935-021-02396-8 34952583 PMC8709944

[B38] Médale-Giamarchi C. Lajoie-Mazenc I. Malissein E. Meunier E. Couderc B. Bergé Y. (2013). RhoB modifies estrogen responses in breast cancer cells by influencing expression of the estrogen receptor. Breast Cancer Res. 15, R6–R13. 10.1186/bcr3377 23339407 PMC3672819

[B39] Milczarek M. (2020). The premature senescence in breast cancer treatment strategy. Cancers 12, 1815. 10.3390/cancers12071815 32640718 PMC7408867

[B40] Munir M. T. Kay M. K. Kang M. H. Rahman M. M. Al-Harrasi A. Choudhury M. (2021). Tumor-associated macrophages as multifaceted regulators of breast tumor growth. Int. J. Mol. Sci. 22, 6526. 10.3390/ijms22126526 34207035 PMC8233875

[B41] Nisar M. Paracha R. Z. Arshad I. Adil S. Zeb S. Hanif R. (2021). Integrated analysis of microarray and RNA-Seq data for the identification of hub genes and networks involved in the pancreatic cancer. Front. Genet. 12, 663787. 10.3389/fgene.2021.663787 34262595 PMC8273913

[B42] Pertea M. Kim D. Pertea G. M. Leek J. T. Salzberg S. L. (2016). Transcript-level expression analysis of RNA-seq experiments with HISAT, StringTie and Ballgown. Nat. Protoc. 11, 1650–1667. 10.1038/nprot.2016.095 27560171 PMC5032908

[B43] Rodgers K. M. Udesky J. O. Rudel R. A. Brody J. G. (2018). Environmental chemicals and breast cancer: an updated review of epidemiological literature informed by biological mechanisms. Environ. Res. 160, 152–182. 10.1016/j.envres.2017.08.045 28987728

[B44] Roemhild K. Von Maltzahn F. Weiskirchen R. Knüchel R. Von Stillfried S. Lammers T. (2021). Iron metabolism: pathophysiology and pharmacology. Trends Pharmacol. Sci. 42, 640–656. 10.1016/j.tips.2021.05.001 34090703 PMC7611894

[B45] Rostovskaya M. Andrews S. Reik W. Rugg-Gunn P. J. (2022). Amniogenesis occurs in two independent waves in primates. Cell Stem Cell 29, 744–759.e6. 10.1016/j.stem.2022.03.014 35439430 PMC9627701

[B46] Sajnani K. Islam F. Smith R. A. Gopalan V. Lam A. K.-Y. (2017). Genetic alterations in Krebs cycle and its impact on cancer pathogenesis. Biochimie 135, 164–172. 10.1016/j.biochi.2017.02.008 28219702

[B47] Sawaki K. Kanda M. Umeda S. Miwa T. Tanaka C. Kobayashi D. (2019). Level of melanotransferrin in tissue and sera serves as a prognostic marker of gastric cancer. Anticancer Res. 39, 6125–6133. 10.21873/anticanres.13820 31704840

[B48] Segovia-Mendoza M. Morales-Montor J. (2019). Immune tumor microenvironment in breast cancer and the participation of estrogen and its receptors in cancer physiopathology. Front. Immunol. 10, 348. 10.3389/fimmu.2019.00348 30881360 PMC6407672

[B49] Shuai C. Yang X. Pan H. Han W. (2020). Estrogen receptor downregulates expression of PD-1/PD-L1 and infiltration of CD8+ T cells by inhibiting IL-17 signaling transduction in breast cancer. Front. Oncol. 10, 582863. 10.3389/fonc.2020.582863 33102239 PMC7545792

[B50] Shumate A. Wong B. Pertea G. Pertea M. (2022). Improved transcriptome assembly using a hybrid of long and short reads with StringTie. PLoS Comput. Biol. 18, e1009730. 10.1371/journal.pcbi.1009730 35648784 PMC9191730

[B51] Sung H. Ferlay J. Siegel R. L. Laversanne M. Soerjomataram I. Jemal A. (2021). Global cancer statistics 2020: GLOBOCAN estimates of incidence and mortality worldwide for 36 cancers in 185 countries. CA a cancer J. Clin. 71, 209–249. 10.3322/caac.21660 33538338

[B52] Thu K. Soria-Bretones I. Mak T. Cescon D. (2018). Targeting the cell cycle in breast cancer: towards the next phase. Cell Cycle 17, 1871–1885. 10.1080/15384101.2018.1502567 30078354 PMC6152498

[B53] Tung K.-F. Pan C.-Y. Chen C.-H. Lin W.-C. (2020). Top-ranked expressed gene transcripts of human protein-coding genes investigated with GTEx dataset. Sci. Rep. 10, 16245. 10.1038/s41598-020-73081-5 33004865 PMC7530651

[B54] Vitting-Seerup K. Sandelin A. (2017). The landscape of isoform switches in human cancers. Mol. Cancer Res. 15, 1206–1220. 10.1158/1541-7786.MCR-16-0459 28584021

[B55] Wang J. Xu Y. Li L. Wang L. Yao R. Sun Q. (2017). FOXC 1 is associated with estrogen receptor alpha and affects sensitivity of tamoxifen treatment in breast cancer. Cancer Med. 6, 275–287. 10.1002/cam4.990 28028927 PMC5269562

[B56] Wang Z. Jiang Q. Dong C. (2020). Metabolic reprogramming in triple-negative breast cancer. Cancer Biol. Med. 17, 44–59. 10.20892/j.issn.2095-3941.2019.0210 32296576 PMC7142847

[B57] Xie Z. Bailey A. Kuleshov M. V. Clarke D. J. Evangelista J. E. Jenkins S. L. (2021). Gene set knowledge discovery with Enrichr. Curr. Protoc. 1, e90. 10.1002/cpz1.90 33780170 PMC8152575

[B58] Xiong X. Liao X. Qiu S. Xu H. Zhang S. Wang S. (2022). CXCL8 in tumor biology and its implications for clinical translation. Front. Mol. Biosci. 9, 723846. 10.3389/fmolb.2022.723846 35372515 PMC8965068

[B59] Yang X. Coulombe-Huntington J. Kang S. Sheynkman G. M. Hao T. Richardson A. (2016). Widespread expansion of protein interaction capabilities by alternative splicing. Cell 164, 805–817. 10.1016/j.cell.2016.01.029 26871637 PMC4882190

[B60] Yeh M.-H. Tzeng Y.-J. Fu T.-Y. You J.-J. Chang H.-T. Ger L.-P. (2018). Extracellular matrix–receptor interaction signaling genes associated with inferior breast cancer survival. Anticancer Res. 38, 4593–4605. 10.21873/anticanres.12764 30061226

[B61] Yin L. Duan J.-J. Bian X.-W. Yu S.-C. (2020). Triple-negative breast cancer molecular subtyping and treatment progress. Breast Cancer Res. 22, 61–13. 10.1186/s13058-020-01296-5 32517735 PMC7285581

[B62] Yip H. Y. K. Papa A. (2021). Signaling pathways in cancer: therapeutic targets, combinatorial treatments, and new developments. Cells 10, 659. 10.3390/cells10030659 33809714 PMC8002322

[B63] Yuan Z. Chen D. Chen X. Yang H. Wei Y. (2017). Overexpression of trefoil factor 3 (TFF3) contributes to the malignant progression in cervical cancer cells. Cancer Cell Int. 17, 7–13. 10.1186/s12935-016-0379-1 28070169 PMC5216547

[B64] Zhang E.-D. Li C. Fang Y. Li N. Xiao Z. Chen C. (2022). STMN1 as a novel prognostic biomarker in HCC correlating with immune infiltrates and methylation. World J. Surg. Oncol. 20, 301. 10.1186/s12957-022-02768-y 36127700 PMC9487063

[B65] Zheng C. Yu S. (2021). Expression and gene regulatory network of SNHG1 in hepatocellular carcinoma. BMC Med. Genomics 14, 28–10. 10.1186/s12920-021-00878-2 33499863 PMC7836560

[B66] Zhu S.-Y. Yu K.-D. (2022). Breast cancer vaccines: disappointing or promising? Front. Immunol. 13, 190. 10.3389/fimmu.2022.828386 PMC8831788 35154149

