# Supplementary material for: Isoform switching leads to downregulation of cytokine producing genes in estrogen receptor positive breast cancer
Source: Front Genet. 2023 Oct 13;14:1230998. doi: 10.3389/fgene.2023.1230998 (PMC10611502; doi:10.3389/fgene.2023.1230998)
Supplement: Supplementary file 1 [file Table2.DOCX]

**Results**

**Table S1:** Top 10 differentially expressed upregulated genes.

| **Gene Name** | **P-value** | **log2FoldChange** | **Expression** | **Experimental validation through Expression Atlas** |
| --- | --- | --- | --- | --- |
| FOXA1 | 4.44E-16 | 4.03 | UP | [(Eswaran et al., 2013)](https://www.zotero.org/google-docs/?7OOYNF); [(Eswaran et al., 2012; Horvath et al., 2013)](https://www.zotero.org/google-docs/?gLVTmX) |
| RHOB | 2.75E-12 | 1.79 | UP | [(Hart et al., 2015)](https://www.zotero.org/google-docs/?FcRIbl) |
| AR | 4.69E-12 | 2.19 | UP | [(Frietze et al., 2014)](https://www.zotero.org/google-docs/?5ZhbET) |
| CMBL | 5.16E-12 | 1.86 | UP | [(Cunha et al., 2014)](https://www.zotero.org/google-docs/?5buDZM) |
| AGR2 | 4.93E-11 | 4.93 | UP | [(Eswaran et al., 2013)](https://www.zotero.org/google-docs/?beliQe); [(Eswaran et al., 2012; Horvath et al., 2013)](https://www.zotero.org/google-docs/?z5gSNX) |
| ESR1 | 5.81E-11 | 3.32 | UP | [(Eswaran et al., 2013)](https://www.zotero.org/google-docs/?zFcxIG); [(Eswaran et al., 2012; Horvath et al., 2013)](https://www.zotero.org/google-docs/?zJ3NnK) |
| TFF3 | 7.72E-11 | 4.75 | UP | [(Liu et al., 2014)](https://www.zotero.org/google-docs/?SUxrKe) |
| SYBU | 8.24E-11 | 1.91 | UP | [(Frietze et al., 2014)](https://www.zotero.org/google-docs/?QxCtXG) |
| CBLC | 2.37E-10 | 1.67 | UP | [(Hart et al., 2015)](https://www.zotero.org/google-docs/?lz2tWk) |
| DNALI1 | 3.04E-10 | 2.06 | UP | [(Tarallo et al., 2017)](https://www.zotero.org/google-docs/?r02Cvn) |

**Table S2:** Top 10 differentially expressed downregulated genes.

| **Gene Name** | **P-value** | **log2FoldChange** | **Expression** | **Experimental validation through Expression Atlas** |
| --- | --- | --- | --- | --- |
| CENPW | 6.66E-16 | -1.84 | DOWN | [(Best et al., 2015)](https://www.zotero.org/google-docs/?Qm0SFc) |
| EN1 | 2.06E-14 | -2.98 | DOWN | [(Eswaran et al., 2013)](https://www.zotero.org/google-docs/?OiSnyh); [(Eswaran et al., 2012; Horvath et al., 2013)](https://www.zotero.org/google-docs/?r0bBWg) |
| A2ML1 | 2.95E-12 | -2.52 | DOWN | [(Best et al., 2015)](https://www.zotero.org/google-docs/?GFB16v) |
| TMSB15A | 8.07E-12 | -1.95 | DOWN | [(Wobble TRNA Modification and Hydrophilic Amino Acid Patterns Dictate Protein Fate \| Nature Communications, n.d.)](https://www.zotero.org/google-docs/?4dJ2qM) |
| FOXC1 | 1.47E-11 | -2.20 | DOWN | [(Eswaran et al., 2013)](https://www.zotero.org/google-docs/?C2svg7); [(Eswaran et al., 2012; Horvath et al., 2013)](https://www.zotero.org/google-docs/?dg0d8X) |
| KRT16 | 3.93E-11 | -2.63 | DOWN | [(Eswaran et al., 2013)](https://www.zotero.org/google-docs/?uL7tS5); [(Eswaran et al., 2012; Horvath et al., 2013)](https://www.zotero.org/google-docs/?qZHD4T) |
| SLC7A5 | 3.98E-11 | -2.18 | DOWN | [(Eswaran et al., 2013)](https://www.zotero.org/google-docs/?BNcyPd); [(Eswaran et al., 2012; Horvath et al., 2013)](https://www.zotero.org/google-docs/?PrLgYr) |
| CDK6 | 5.90E-11 | -1.60 | DOWN | [(Catalano et al., 2019)](https://www.zotero.org/google-docs/?Gq6Cp8) |
| MELTF | 1.66E-10 | -1.85 | DOWN | [(Best et al., 2015)](https://www.zotero.org/google-docs/?qu5qUk) |
| CA9 | 2.41E-10 | -2.45 | DOWN | [(Best et al., 2015)](https://www.zotero.org/google-docs/?91xZ9E) |

**Table S3:** Top 3 downregulated immune genes that have isoforms significantly used in ERP

| **Gene name** | **P-value** | **LogFC** | **Expression** | **Experimental validation through Expression Atlas** |
| --- | --- | --- | --- | --- |
| MELTF | 1.66E-10 | -1.85184 | Down | [(Best et al., 2015)](https://www.zotero.org/google-docs/?Z6rvkZ) |
| STMN1 | 4.25E-08 | -1.53543 | Down | [(Best et al., 2015)](https://www.zotero.org/google-docs/?D7Xxoe) |
| CXCL8 | 5.51E-05 | -1.97224 | Down | [(Eswaran et al., 2012, 2013; Horvath et al., 2013)](https://www.zotero.org/google-docs/?cBplsH) |


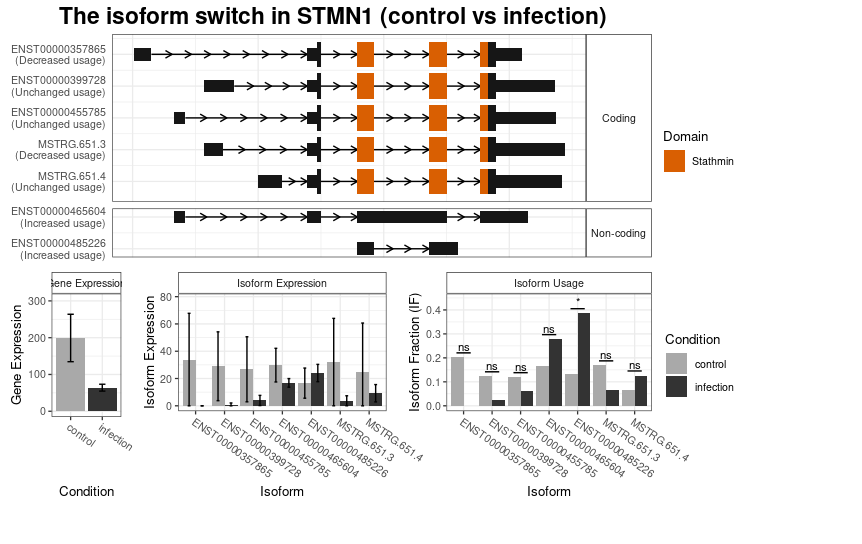


**Figure S1:** Isoform switching of differentially expressed immune genes. ‘*’ represents significant isoform usage. Significant switches in isoform usage is represented by STMN1 across ERP vs. TNBC. By comparing the isoform usage across conditions, it was revealed that STMN1 has a single isoform (ENST00000485226) which was overexpressed in ERP


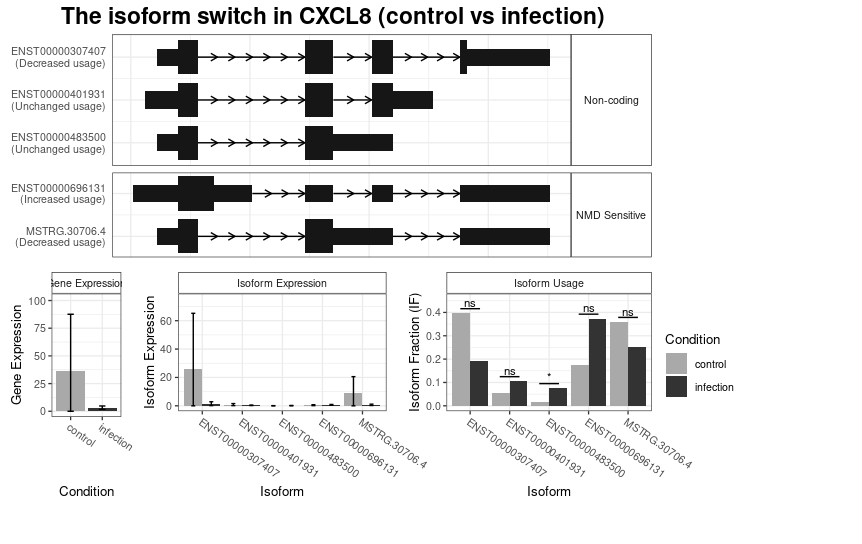


**Figure S2:** Isoform switching of differentially expressed immune genes. ‘*’ represents significant isoform usage. By comparing the isoform usage across conditions, it was revealed that CXCL8 also has one isoform (ENST00000483500) which was significantly used in ERP


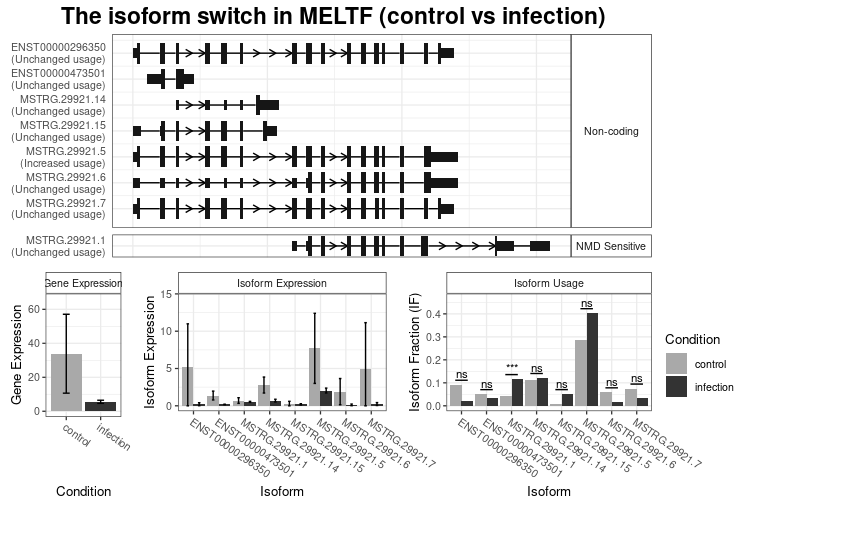


**Figure S3:** Isoform switching of differentially expressed immune genes. ‘*’ represents significant isoform usage. By comparing the isoform usage across conditions, it was found that a novel isoform (MSTRG.29921.1) of MELTF was overexpressed in ERP
